# Variability in Prevalence of *Helicobacter pylori* Strains Resistant to Clarithromycin and Levofloxacin in Southern Poland

**DOI:** 10.1155/2012/418010

**Published:** 2012-05-27

**Authors:** Elżbieta Karczewska, Karolina Klesiewicz, Iwona Skiba, Izabela Wojtas-Bonior, Edward Sito, Krzysztof Czajecki, Małgorzata Zwolińska-Wcisło, Alicja Budak

**Affiliations:** ^1^Department of Pharmaceutical Microbiology, Jagiellonian University Medical College, Medyczna 9, 30-688 Krakow, Poland; ^2^Outpatient Clinic of Gastroenterology Falck Medycyna 30-036, Krakow, Poland; ^3^Department of Gastroenterology, Hepatology and Infectious Diseases, Jagiellonian University Medical College, Śniadeckich 5, 31-531 Krakow, Poland

## Abstract

*Background*. An increasing resistance of *Helicobacter pylori *strains to antimicrobial agents is the serious therapeutic problem. The aim of this study was to compare the primary and secondary resistance of *H. pylori* strains isolated between 2006–2008 (data published) and 2009–2011 to clarithromycin and levofloxacin. *Material and Methods*. 220 dyspeptic patients (153 before treatment, 67 after), were enrolled in the study. 51 *H. pylori *strains were isolated. MIC values of clarithromycin and levofloxacin were determined by the *E*-test method. The statistical analysis was conducted with the *χ* ^2^ test with Yates correction at the 0.05 significance level (*P* ≤ 0.05). *Results*. Between 2006 and 2008, 34% (39/115) of *H. pylori* strains were resistant to clarithromycin (primary 21% (19/90), secondary 80% (20/25)). 5% (6/115) of strains were resistant to levofloxacin (primary 2% (2/90), secondary 16% ((4/25); data published) Between 2009–2011, 22% (11/51) of *H. pylori *strains were resistant to clarithromycin (primary 19% (8/43), secondary 38% (3/8)). 16% (8/51) of strains were resistant to levofloxacin (primary 12% (5/43), secondary 38% (3/8)). *Conclusion*. The present study has shown the increasing amount of resistant *H. pylori *strains isolated from patients in Southern Poland to levofloxacin and decreasing number of resistant strains to clarithromycin.

## 1. Introduction

*Helicobacter pylori (H. pylori) *is a Gram-negative, microaerophilic, and urease-positive spiral shaped bacterium, which colonizes the gastric mucosa of 50% of the population worldwide [1, 2]. The incidence of the infection is associated mostly with childhood as well as socioeconomic and sanitary conditions. *Helicobacter pylori* infection plays a major role in peptic ulcer disease, low-grade mucosa-associated lymphoid tissue (MALT) lymphoma, and gastric cancer. Thanks to the discovery of this pathogen by Marshall and Warren in 1982, peptic ulcer diseases are no longer chronic but can be cured by the regimen of antibiotics and gastric antisecretory drugs [3].

 The preferred eradication therapy is triple or quadruple therapy, which is combined therapy including three types of drugs: antisecretory drugs, cytoprotectants, and antibiotics and chemotherapeutic drugs. Current guidelines from the American College of Gastroenterology and the European Helicobacter Study Group (EHSG) recommend a clarithromycin-based triple therapy for the first 5 days (a proton pump inhibitor (PPI) plus amoxicillin and clarithromycin) or a bismuth quadruple therapy (a PPI plus bismuth, metronidazole and tetracycline) [4, 5]. Obligatory procedures for the management of *H. pylori *infection in Poland elaborated upon by the Working Group of the Polish Society of Gastroenterology (PTG) are based on new guidelines from the Third Maastricht Consensus Conference in 2005 [6].

Current regimens of treatment *H. pylori* infection in Poland are as follows.

- The First-Line Treatment. PPI, amoxicillin (1000 mg), and metronidazole (500 mg) twice a day, 10–14 days, and PPI, clarithromycin (500 mg), and metronidazole (500 mg) twice a day, 10–14 days, or PPI, amoxicillin (500 mg), and clarithromycin (500 mg) twice a day, 10–14 days.

- The Second-Line Treatment. PPI, amoxicillin (1000 mg), and metronidazole (500 mg) twice a day and tetracycline (250 mg) three times daily prolonged to 14 days, or PPI, amoxicillin (1000 mg), and metronidazole (500 mg) twice a day and bismuth salts (120 mg) four times daily; prolonged to 14 days.

- The Third-Line Treatment. Evaluation of the susceptibility of the strains to the currently used antimicrobial agents: amoxicillin, metronidazole, clarithromycin, and tetracycline; possible introduction of levofloxacin; adding a probiotic [6].

Recommendations of PTG were published in 2008 and were the first polish recommendations which allow introduction of levofloxacin in treatment of *H. pylori* infection.

The increasing level of antibiotic resistance in *H. pylori *strains had a drastic effect on the successful treatment [7, 8]. The most recent Maastricht guidelines recommend substituting metronidazole for clarithromycin in case where the resistance level exceeds 15–20% [9]. However, according to the Maastricht recommendation, if the resistance level to metronidazole exceeds 40% and for clarithromycin 15–20%, these antimicrobial agents should not be used or susceptibility testing should be done. In addition, it recommends local permanent monitoring of* H. pylori *susceptibility to antimicrobial agents [5]. Emerging evidence indicates that resistance rates to metronidazole could constitute the real problem. On the other hand, some scientists believe that the resistance might be overcome with increased doses of metronidazole [10]. The rate of clarithromycin resistance is increasing, and one of the reasons of this increase is likely to be a greater use of clarithromycin in the treatment of respiratory tract infections in the community. Clarithromycin resistance in *H. pylori *is associated with treatment failure, although geographical variations were also observed [7, 11]. In Poland the resistance of *H. pylori *to antimicrobial drugs used in the therapy is high and amounts to 28% to clarithromycin (primary resistance 22%, secondary resistance 54%) and 46% to metronidazole (primary resistance 41%, secondary resistance 68%) (data published by PTG) [6, 12]. Therefore, in accordance with the Maastricht recommendations, in Poland clarithromycin and metronidazole should not be used without previous susceptibility testing [5].

 When the first-line therapy is unsuccessful, we need the effective second-line therapy. Evolving research has demonstrated that the introduction of new drugs, such as levofloxacin and rifabutin, provides new possibilities of treatment [7, 10, 11]. However, the current recommendation of PTG is to entertain the introduction of levofloxacin as the third-line empirical treatment [6]. Nevertheless, some studies carried out by Molina-Infante in Spain examined the introduction of levofloxacin in the first-line treatment in triple and sequential regimens and demonstrated the advantage of levofloxacin in both combinations. Levofloxacin may be a good alternative to clarithromycin in the region with high percentage of resistant *H. pylori* strains to clarithromycin. [7, 11]. As a result of frequent resistance of *H. pylori* to clarithromycin in Poland and recommendations of PTG (2008) that enable the introduction of levofloxacin to* H. pylori* eradication therapy, many physicians have started using the levofloxacin in first-line treatment (data not published).

 Levofloxacin, a bactericidal fluoroquinolone of the 3rd generation antibiotics, has also the activity in the second-line therapy. Levofloxacin may be used as a substitute for clarithromycin in either a standard triple or sequential regimen. A large study comparing antibiotics in either of regimens shows a clear advantage to levofloxacin in both combinations. It has been proposed that levofloxacin-based regimens are the most beneficial in areas where clarithromycin resistance is higher [13–16]. The introduction of levofloxacin to the treatment scheme raises many hopes, but the resistance to levofloxacin is a growing problem in Spain (from 6% to more than 25% over the last 5 years) [17]. A rapidly increasing rate of fluoroquinolone resistance was reported in several countries [7]. The apparently rapid rate at which fluoroquinolone resistance seems to develop may limit the use of levofloxacin in *H. pylori *eradication to the second-line therapy.

 Since the resistance to antimicrobials is a major cause of eradication failure, the monitoring of antimicrobial resistance of *H. pylori *in each domestic area should be warranted, especially for clarithromycin and the commonly applied metronidazole. Such monitoring is also recommended by the Maastricht III Consensus. For developing countries this monitoring should probably also include other antimicrobials used in the eradication therapy [18–20]. Therefore, the aim of this prospective study was to assess the primary and secondary resistance of *H. pylori *strains isolated from adult patients, from the Malopolska region in Poland between 2006–2008 [13] and 2009–2011, to antibacterial drugs (clarithromycin and levofloxacin) used clinically for *H. pylori* eradication.

## 2. Materials and Methods

### 2.1. Patients

The study enrolled a group of 220 dyspeptic patients aged 16–87, who underwent gastroscopy in the “Falck” Health Care Center in Krakow, Poland.

153 patients had never been treated for *H. pylori* infection, whereas 67 patients underwent the *H. pylori* eradication therapy.

The plan of the study was approved by the Bioethical Commission of the Jagiellonian University, and each patient signed the informed consent for the participation in the study.

### 2.2. Clinical Material

 During gastroscopy two biopsy specimens (bioptates) were taken from each patient. Bioptates were collected from the antrum and the body of the stomach. Bioptates were transferred in a transportation medium, Portagerm pylori (bioMérieux, Marcy-l'Etoile, France), and then sent for microbiological tests, which were performed at the Department of the Pharmaceutical Microbiology of the Jagiellonian University Medical College.

### 2.3. Bacterial Culture and Susceptibility Testing

 Bioptate was homogenized in glass sterile mortars to ensure a homogeneous distribution of bacteria in the whole specimen. Homogenate was inoculated onto the solid medium, Schaedler agar with 5% sheep blood added (bioMérieux, Marcy-l'Etoile, France) and medium, Schaedler agar with 5% sheep blood, and Dent selective supplement added (Helicobacter pylori Selective Supplement-DENT, Oxoid, Basingstoke, UK). The culture was carried out for 3 to 7 days under 5% CO_2_ at 37°C.

 The presence of *H. pylori* in the tested material was confirmed by the visual examination of the typical colonies morphology on the plate with medium, positive biochemical tests for catalase, oxidase, and urease. Furthermore, Gram-staining preparation from the colony was performed to confirm the presence of Gram-negative spiral bacteria.

 The susceptibility of *H. pylori* strains to antimicrobial agents was assessed by the quantitative method, *E*-test (AB Biodisk, Solna, Sweden), which determined the minimal inhibitory concentration (MIC) of the drug that inhibits the growth of bacterial strains. The susceptibility to clarithromycin and levofloxacin was tested for each *H. pylori* strain. From the pure *H. pylori* culture, one colony was taken to prepare the suspension in 0.85% NaCl on an equivalent of 3.0 McFarland units. The inoculum was spread on the plate with the Schaedler agar with 5% sheep blood (bioMérieux, Marcy-l'Etoile, France) within 15 minutes after the preparation. Then, *E*-test stripes with the clarithromycin and levofloxacin gradient were placed on plates according to manual of the manufacturer (AB Biodisk, *E*-test technical manual), separately for clarithromycin and levofloxacin. Plates were incubated in microaerophilic conditions at 37°C for 72 hrs.

 The breakpoints used to qualify strains as resistant according to the MIC values were 1 mg/L for both tested antibiotics, as previously described [21, 22].

The determination of MIC values was carried out against the reference *H. pylori* strain from the American Type Culture Collection, ATCC 43504 *Helicobacter pylori*, to ensure the quality of susceptibility tests.

### 2.4. Statistical Analysis

The statistical parameters such as: mean values and chi-squared test of Independence (*χ* ^2^ test) were performed. The accepted significance level was 0.5 (results with *P* ≤ 0.05 were considered statistically significant). In cases where the expected values were less than 5, the Yates correction was used.

The association between the primary and the secondary *H. pylori* resistance to the tested antibiotics was checked.

Moreover, the statistical analysis tested the differences between the level of primary and secondary *H. pylori* resistance to clarithromycin and levofloxacin in the years of our study (2009–2011) and the previous study which was carried out in the years 2006–2008, also in our Department and showed the level of *H. pylori* resistance in the same region of Southern Poland, Malopolska [13].

## 3. Results and Discussion

### 3.1. Results

**Figure 1:**
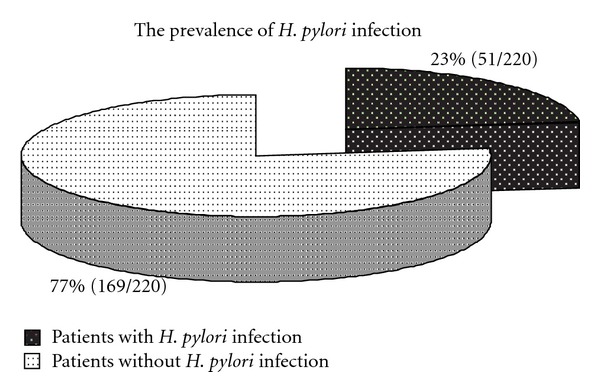
The prevalence of *H. pylori* infection among dyspeptic patients enrolled in the study in 2009–2011.

Among 220 patients with dyspeptic symptoms admitted to the study between January 2009 and December 2011, the presence of *H. pylori* infections was confirmed in 51 cases. The prevalence of *H. pylori* infections among dyspeptic patients in Southern Poland amounted to 23% (51/220 Figure 1). The presence of *H. pylori* was confirmed by CLO test—rapid urease test—performed by a doctor and bacterial culture.

51 strains of *H. pylori* were successfully isolated from biopsy specimens of 51 patients who were identified as positive for *H. pylori*. The group of *H. pylori-*positive patients consisted of 28 women (55%) and 23 men (45%), which indicates that both ganders were equally represented in the study. The average age of this group of patients was 45.6 years (aged 18–75 years).

**Figure 2:**
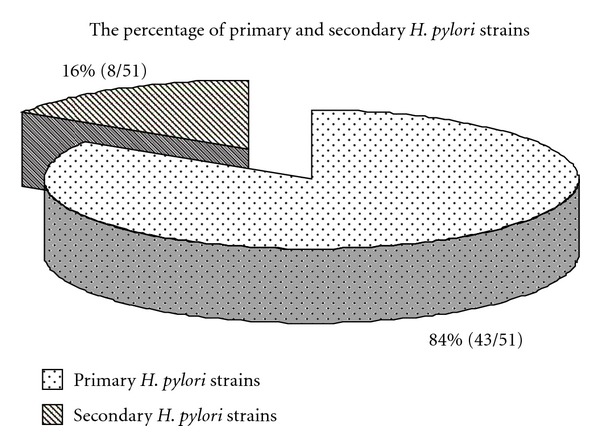
The percentage of primary and secondary *H. pylori* strains isolated from dyspeptic patients enrolled in the study in 2009–2011.

 In total, 43 strains were derived from patients who had never been treated for *H. pylori* infections (primary strains 84%) and 8 strains were derived from patients after the failed therapy (secondary strains 16%) (Figure 2).

Susceptibility to clarithromycin and levofloxacin was tested for all *H. pylori* strains by the quantitative method, *E*-test. The obtained MIC values ranged from 0.016 to 12 mg/L for clarithromycin and from 0.012 to 32 mg/L for levofloxacin. Mean MIC values were as follows: 1.22 mg/L for clarithromycin and 1.42 mg/L for levofloxacin.

In total, in the years 2009–2011, the ratio of *H. pylori* strains susceptible to clarithromycin amounted to 78% (40/51), while the ratio of resistant strains amounted to 22% (11/51); primary resistance was 19% (8/43 strains) and secondary 38% (3/8 strains). The ratio of *H. pylori* strains susceptible to levofloxacin amounted to 84% (43/51 strains), while the ratio of resistant strains amounted to 16% (8/51 strains); primary resistance 12% (5/43), secondary 38% (3/8) (Table 1).

In the years 2006–2008, 115 strains were isolated. 34% (39/115) of *H. pylori* strains were resistant to clarithromycin (primary 21% (19/90), secondary 80% (20/25)). 5% (6/115) of strains were resistant to levofloxacin (primary 2% (2/90), secondary 16% (4/25)) [13].

The comparison of the *H. pylori*-resistant strains to clarithromycin and levofloxacin, isolated between 2006–2008 [13] and 2009–2011, was conducted with the use of the *χ* ^2^ test. An increase of the amount of resistant strains to levofloxacin was statistically significant; 5% (6/115) between 2006 and 2008 [13] versus 16% (8/51) between 2009 and 2011, *P* = 0.05 (with the Yates correction).

**Figure 3:**
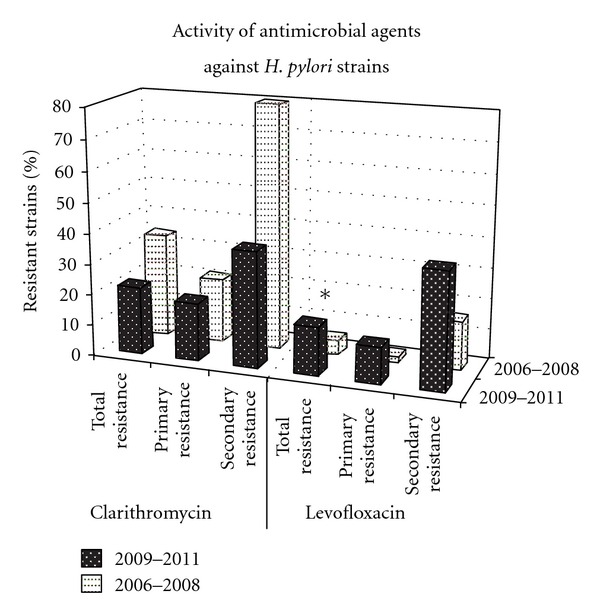
Activity of clarithromycin and levofloxacin against primary and secondary *H. pylori* strains. *statistically significant differences between the level of resistance to levofloxacin in the years 2006–2008 and 2009–2011.

 Nevertheless, the amount of *H. pylori*-resistant strains to clarithromycin is decreasing. The total amount of resistant strains decrease from 34% in 2006–2008 [13] to 22% in 2009–2011; however it is statistically insignificant (*P* = 0.16 (Table 2, Figure 3)).

## 4. Discussion

Variations of the prevalence of resistant *H. pylori* strains depend on some factors, for instance, the use of antibiotics and chemotherapeutics in recommended patterns of antimicrobial agents, and are geographically differentiated [23].

The resistance of *H. pylori* strains to levofloxacin is quickly acquired; thus, it is the growing problem [7, 23]. For example, in France it increased from 3.3% in 1999 to 17.5% in 2003 [21]; in Spain the resistance increased from 6% to more than 25% over the last five years [17, 24]. In another country, such as Iran, the resistance of *H. pylori *to fluoroquinolones has also been increasing although it had not been reported before—primary resistance has amounted to 5.3% for levofloxacin [18].

Our study has shown that in Poland there is also a significant increase of *H. pylori* strains resistant to levofloxacin, from 5% in 2006–2008 [13] to 16% in 2009–2011 (*P* = 0.05). Many studies have shown that resistance to fluoroquinolones is easily acquired and is due to point mutations in gyrA genes [21, 23, 25, 26]. The higher rate of *H. pylori*-resistant strains may be caused by the more frequent use of levofloxacin in the treatment of *H. pylori* infections. Studies conducted in Belgium over the last 20 years (1990–2009) show the correlation between consumption of antibiotics and the rates of resistant *H. pylori* strains [27]. Also another study, carried out by Cabrita et al. in Portugal, shows the correlation between increased use of antibiotics and the growth in prevalence of resistant *H. pylori* strains to these antibiotics [28]. Nevertheless, there is no commonly available information about usage of antibiotics and chemotherapeutics in outpatient clinic in Poland, but, as known, fluoroqinolones are used not only in *H. pylori *infection but also in treatment of infections of genitourinary tract and respiratory tract, gastrointestinal diseases, infection of skin and soft tissues, and many others [25, 29, 30]. This usage of fluoroquinolones and cited studies allows to conclude that increasing resistance of *H. pylori *strains to levofloxacin in Southern Poland may be caused by more common use of levofloxacin and other fluoroquinolones in community and also in treatment of *H. pylori* infections. Susceptibility testing has not been routinely performed and anti-*H. pylori* drugs like levofloxacin are used in the empirical therapy as suggested by many researchers. However, due to the fact that the resistance to levofloxacin is quickly acquired, susceptibility testing should be routinely carried out to enable properly selecting treatment model, or levofloxacin should not be used commonly but only in the rescue third-line therapy, when treatment with clarithromycin and metronidazole failed (as it is recommended by EHSG and PTG [5, 6]) to avoid the further increase of resistance of *H. pylori* to antimicrobial agents [31]. Moreover, Marzio et al. dealt with the role of preliminary susceptibility testing before therapy and after failed therapy. It has been suggested that triple therapy with levofloxacin, amoxicillin, and PPI should not be used without previous susceptibility test in the region where primary resistance of *H. pylori* to levofloxacin amounted to 10% [32]. In our study, 16% of *H. pylori* strains was resistant to levofloxacin and primary resistance 12%.

According to EHSG and the Polish Society of Gastroenterology recommendations, there are three schemes of treatment which suggested the use of levofloxacin as the third-line treatment [5, 6]. Moreover, several studies which showed the efficacy of the third-line rescue therapy with levofloxacin were carried out [31, 33, 34]. Furthermore, levofloxacin was also successfully tested as a good substitute of clarithromycin in the area with the high prevalence of clarithromycin-resistant *H. pylori* strains [7, 35] and as a good alternative for patients allergic to penicillin [36].

Positive results of these studies were likely to contribute to the increased use of levofloxacin instead of clarithromycin in the empirical treatment. Apart from that, fluoroquinolones as drugs with a broad spectrum of activity against bacteria are commonly used in the treatment of many diseases, not only in the treatment of *H. pylori* infections.

An interesting result shown by our research is the change in the profile of the susceptibility of *H. pylori* strains isolated from patients in Southern Poland to clarithromycin. The resistance to clarithromycin decreased in comparison to the previous years 2006–2008. The current level of resistance of *H. pylori* to clarithromycin has amounted to 22%, while in 2006–2008 it was equal to 34% [13]. This change may be caused by the lower consumption of this antimicrobial agent and higher consumption of levofloxacin instead of clarithromycin. This proposal is due to the changes in the profile of *H. pylori* susceptibility and the previously cited studies indicating the relationship between the amount of drug consumption and the amount of resistance of *H. pylori* strains to this drug [27]. It is a hypothesis which would require further detailed research and analysis. However, as the Maastricht III Consensus Report recommended, we carry out the monitoring of antibiotics resistance of *H. pylori* strains in our region of Poland—Southern Poland.

Interesting results have been obtained in Brazil, the research shows that the resistance to clarithromycin is lower than that to levofloxacin (8% versus 23%), which suggests that clarithromycin is still a good option in the treatment of *H. pylori* infections [16]. If the level of resistance to levofloxacin continues to rise and the downward trend of resistance to clarithromycin is sustained, a similar situation may occur in Poland.

## 5. Conclusion

All things considered, it should be noted that the resistance of *H. pylori* strains is changing and depends on commonly used antimicrobial agents, so the obligatory susceptibility testing before the treatment would be a much better solution to avoid the further increase of resistance of *H. pylori* and other bacteria to antibiotics commonly used in treatment of *H. pylori* infection [31]. Moreover, the present study shows rapidly increasing resistance of *H. pylori* strains isolated from patients in Poland, to levofloxacin. That could discourage the use of this fluoroquinolone in the empirical first-line therapy of *H. pylori* infections and suggest that it should be avoided to overuse of levofloxacin as a first-line therapy. Thus, *H. pylori* resistance to clarithromycin should be permanently monitored due to the variability of the prevalence of resistant *H. pylori* strains.

## Figures and Tables

**Table 1:** Comparison of resistance of *H. pylori* primary and secondary strains to clarithromycin and levofloxacin in 2009–2011.

Antimicrobial agent	No. (%) of resistant *H. pylori* strains in the		
	years 2009–2011		
	All strains	Primary strains	Secondary strains
	*n* = 51	*n* = 43	*n* = 8
CLA^(1)^	11 (22%)	8 (19%)	3 (38%)
LEV^(1)^	8 (16%)	5 (12%)	3 (38%)

^(1)^CLA: clarithromycin, LEV: levofloxacin.

**Table 2:** Comparison of resistance of *H. pylori* strains to clarithromycin and levofloxacin between 2006–2008 [13] and 2009–2011.

Antimicrobial agent	No. (%) of *H. pylori*-resistant strains		
	2006–2008 [13]	2009–2011	*P* value^(1)^
	*n* = 115	*n* = 51	
CLA^(2)^	39 (34%)	11 (22%)	0,16 NS^(3)^
LEV^(2)^	6 (5%)	8 (16%)	0,05^(4)^

^(1)^ *P* value (chi-square test) with the Yates correction. *P*≤ 0.05 was deemed statistically significant.

^(2)^CLA: clarithromycin, LEV: levofloxacin.

^(3)^NS: non significant.

^(4)^Statistically significant differences between the level of resistance in the years 2006–2008 and 2009–2011.
